# Ethnic Disparities in Achieving Treatment Targets and Organ Damage Accrual in Systemic Lupus Erythematosus: A Multi-Centre Study from Malaysia

**DOI:** 10.3390/jcm15093387

**Published:** 2026-04-29

**Authors:** Syahrul Sazliyana Shaharir, Teh Cheng Lay, Asmahan Mohamed Ismail, Azwarina Hanim Ramlan, Tan Chou Luan, Hairul Hadi Ariff, Anna Farazilah Mohd Salleh, Ng Chun Ruh, Asmah Mohd, Chua Siew Houy, Nadiah Mohd Noor, Ling Guo Ruey, Sharifah Aishah Wan Mohamad Akbar, Raja Jasmin Begum Raja Mohamed, Fariz Yahya, Kan Sow Lai, Wan Syamimee Wan Ghazali, Shakira Selvananda, Dayang Masyrinartie Suhaili, Noraini Mat Husin, Hashimah Abu Mansor Matardiah, Eashwary Mageswaran, Suhaida Ahmad Maulana, Mariam Hamid Mustapha, Wan Rosmaiza Wan Musa, Nor Shuhaila Shahril, Liza Mohd Isa, Shereen Ch’ng Suyin, Norliza Zainudin, Mollyza Mohd Zain, Habibah Mohd Yusoof, Chong Hwee Cheng, Hong Hooi Chuen, Jasmine Yew Sze Yin, Siti Mariam Ab Rahim, Lim Shiau Li, Gan Syang Pyng, Hazlyna Baharuddin, Nur Farhana Abdul Manaf, Malehah Mohd Noh, Sakthiswary Rajalingham, Mohd Shahrir Mohamed Said, Rozita Mohd, Muhammad Irfan Abdul Jalal

**Affiliations:** 1 Faculty of Medicine, Universiti Kebangsaan Malaysia, Cheras 56000, Wilayah Persekutuan Kuala Lumpur, Malaysia; sakthiswary@ukm.edu.my (S.R.); drshahrir70@gmail.com (M.S.M.S.); rozi8286@gmail.com (R.M.); 2 Department of Medicine, Sarawak General Hospital, Kementerian Kesihatan Malaysia, Jalan Hospital, Kuching 93586, Sarawak, Malaysia; tehchenglay@yahoo.com (T.C.L.); shaishah80@yahoo.com (S.A.W.M.A.); 3 Department of Medicine, Hospital Raja Perempuan Zainab II, Kementerian Kesihatan Malaysia, Bandar, Kota Bharu 15586, Kelantan, Malaysia; asma_dr_2000@yahoo.co.uk; 4 Department of Medicine, Hospital Sultanah Bahiyah, Kementerian Kesihatan Malaysia, Km 6, Jln Langgar, Alor Setar 05460, Kedah, Malaysia; azwahanim79@yahoo.com (A.H.R.); chouluan@gmail.com (T.C.L.); 5 Department of Medicine, Hospital Putrajaya, Kementerian Kesihatan Malaysia, Pusat Pentadbiran Kerajaan Persekutuan, Presint 7, Putrajaya 62250, Kuala Lumpur, Malaysia; rosmaiza@yahoo.com (W.R.W.M.); drshu73@gmail.com (N.S.S.); lizamisa@gmail.com (L.M.I.); 6 Department of Medicine, Hospital Queen Elizabeth, Kementerian Kesihatan Malaysia, 13a, Jalan Penampang, Kota Kinabalu 88200, Sabah, Malaysia; hardy_umalaya@hotmail.com (H.H.A.); halizaraf@hotmail.com (A.F.M.S.); 7 Department of Medicine, Hospital Sultanah Fatimah, Kementerian Kesihatan Malaysia, Jln Salleh, Taman Utama Satu, Muar 84000, Johor, Malaysia; angiepaeds@gmail.com (N.C.R.); nadiahmnoor@gmail.com (N.M.N.); 8 Department of Medicine, Hospital Tuanku Ja’afar, Kementerian Kesihatan Malaysia, Jalan Rasah, Bukit Rasah, Seremban 70300, Negeri Sembilan, Malaysia; asmahmohd@yahoo.com (A.M.); siewhouy@gmail.com (C.S.H.); 9 Faculty of Medicine, IMU University, Bukit Jalil 57000, Wilayah Persekutuan Kuala Lumpur, Malaysia; 10 Department of Medicine, Hospital Sibu, Kementerian Kesihatan Malaysia, Batu 5 1/2, Jalan Ulu Oya, Pekan Sibu, Sibu 96000, Sarawak, Malaysia; johnlinggr2005@yahoo.com.sg; 11 Faculty of Medicine, Nursing and Health Sciences, SEGi University, Jalan Ulu Oya, Sibu 96000, Sarawak, Malaysia; 12 Faculty of Medicine, Universiti Malaya, Kuala Lumpur 50603, Wilayah Persekutuan Kuala Lumpur, Malaysia; rjasmin@ummc.edu.my (R.J.B.R.M.); drfarizyahya@gmail.com (F.Y.); 13 Department of Medicine, Hospital Bukit Mertajam, Kementerian Kesihatan Malaysia, Jln Kulim, Bukit Mertajam 14000, Pulau Pinang, Malaysia; kansowlai@yahoo.com; 14 School of Medical Sciences, Hospital Universiti Sains Malaysia, Kubang Kerian 16150, Kelantan, Malaysia; mimeeghazali@yahoo.com; 15 Department of Medicine, Hospital Tengku Ampuan Afzan, Kementerian Kesihatan Malaysia, Jalan Tanah Putih, Kuantan 25100, Pahang, Malaysia; shakiraselva@gmail.com (S.S.); dayangsuhaili@yahoo.com (D.M.S.); 16 Department of Medicine, Hospital Raja Permaisuri Bainun, Kementerian Kesihatan Malaysia, Jalan Raja Ashman Shah, Ipoh 30450, Perak, Malaysia; ninimh76@yahoo.com; 17 Department of Medicine, Hospital Tuanku Fauziah, Kementerian Kesihatan Malaysia, 3, Jalan Tun Abdul Razak, Pusat Bandar Kangar, Kangar 01000, Perlis, Malaysia; rubp85@gmail.com; 18 Department of Medicine, Hospital Tengku Ampuan Rahimah, Kementerian Kesihatan Malaysia, Jalan Langat, Klang 41200, Selangor, Malaysia; dresa712@hotmail.com (E.M.); suahmadmaulana@yahoo.com (S.A.M.); mariamhmus@gmail.com (M.H.M.); 19 Department of Medicine, Hospital Selayang, Kementerian Kesihatan Malaysia, Lebuhraya Selayang—Kepong, Batu Caves 68100, Selangor, Malaysia; shereenchg@gmail.com (S.C.S.); niazailis301076@gmail.com (N.Z.); drmollyza@gmail.com (M.M.Z.); habibah_yusoof@yahoo.com (H.M.Y.); 20 Department of Medicine, Hospital Melaka, Kementerian Kesihatan Malaysia, Jalan Mufti Haji Khalil, Melaka City 75400, Melaka, Malaysia; chonghweecheng@yahoo.com (C.H.C.); hhchuen100@hotmail.com (H.H.C.); jasy85158@gmail.com (J.Y.S.Y.); 21 Department of Medicine, Hospital Sultanah Nur Zahirah, Kementerian Kesihatan Malaysia, Jalan Sultan Mahmud, Kuala Terengganu 20400, Terengganu, Malaysia; smaroy2000@yahoo.com; 22 Department of Medicine, Hospital Sultan Idris Shah Serdang, Kementerian Kesihatan Malaysia, Jalan Puchong, Kajang 43000, Selangor, Malaysia; shiauli83@hotmail.com (L.S.L.); gs_pyng@yahoo.com (G.S.P.); 23 Faculty of Medicine, Universiti Teknologi Mara (UiTM), Sungai Buloh 47000, Selangor, Malaysia; hazlynabaharuddin@yahoo.com (H.B.); farhanamanaf@uitm.edu.my (N.F.A.M.); 24 Faculty of Medicine and Health Sciences, Universiti Malaysia Sabah, Kota Kinabalu 88400, Sabah, Malaysia; sitimalehah2@gmail.com

**Keywords:** ethnic, damage, remission, lupus

## Abstract

**Background/Objectives**: Systemic lupus erythematosus (SLE) is a heterogeneous disease with substantial variability in clinical manifestations and outcomes, influenced by ethnic and geographical diversity. Remission is the optimal treatment target, while low lupus disease activity state is an accepted alternative goal. Although sustained remission has been associated with reduced organ damage, the impact of early attainment of treatment targets on subsequent damage accrual in SLE remains incompletely defined. To explore a potential window of opportunity, this study aimed to identify factors associated with achieving remission or low lupus disease activity state within the first year of SLE diagnosis and to examine their associations with organ damage. **Methods**: This retrospective study was conducted across 22 rheumatology centres in Malaysia and included patients with systemic lupus erythematosus (SLE) who had complete follow-up from diagnosis until attainment of treatment targets. Treatment targets were defined as achieving either early remission or a low disease activity state (LDAS). Given the retrospective nature of the study and the unavailability of complete Physician’s Global Assessment data, the definitions of early remission and LDAS were modified by excluding this component. Accordingly, treatment targets were defined as attainment of a clinical SLE Disease Activity Index (cSLEDAI) score of 0 with oral prednisolone doses of ≤5 mg/day and ≤7.5 mg/day, respectively, within 12 months of SLE onset. Multivariable Cox proportional hazards regression and logistic regression analyses were performed to determine factors associated with early remission and organ damage, respectively. **Results**: A total of 1599 patients were included, the majority of whom were female (92.5%). The cohort was predominantly Malay (68.7%), followed by Chinese (17.4%), Indigenous groups (10.8%) and Indian (3.0%). Early attainment of treatment targets was achieved in 45.7% of patients, while 54.3% experienced delayed attainment. In the multivariable cox regression model, the Malay and Chinese ethnic group demonstrated a significantly lower likelihood of achieving early remission compared to the Indigenous ethnic group. Patients with longer SLE duration, low C4 levels, presence of haematological and renal manifestations at the initial presentation, were identified as additional adverse factors associated with lower likelihood of early remission. Overall, 16.9% of patients accrued organ damage. Independent factors associated with organ damage included Indian ethnicity [OR 5.75, 95% CI 1.39–23.81, *p* = 0.02], delayed remission [OR 2.97, 95% CI 1.51–5.83) and absence of baseline hydroxychloroquine therapy [OR 4.13, 95% CI 1.21–14.07; *p* = 0.020]. **Conclusions**: Ethnic disparities were observed in the early attainment of the treatment targets, as well as in organ damage accrual within the Malaysian multi-ethnic SLE cohort. The significant association between the delay in achieving the treatment targets and organ damage underscores the importance of adopting an early treat-to-target approach in SLE management.

## 1. Introduction

Systemic lupus erythematosus (SLE) is an autoimmune disease which is characterised by dysregulation of the immune system, which produces autoantibodies that attack the individual’s own organs [1]. SLE commonly affects young reproductive-age women, and Asian patients (including Malaysians) tend to have the severe form of the disease with higher mortality and morbidity [2]. The reported prevalence of SLE in Malaysia was approximately 50–60/100,000 population [3]. It is believed that the current prevalence may be higher due to the increase in rheumatology services in Malaysia [4] in the recent years.

The heterogeneity of SLE across ethnic groups has been postulated to result from differences in immune pathways linked to the diverse gene loci. Hence, the manifestations of SLE vary across the different ethnic groups in Malaysia leading to the delayed diagnoses of SLE. Malaysian Chinese patients tend to have renal involvement [3], while higher musculoskeletal manifestations were found among Malays [5], and mucocutaneous manifestations were more prevalent among Indian patients [2].

Malaysian SLE patients tend to have more severe disease compared to their Western counterparts. Earlier reports have demonstrated that the involvement of major organs, particularly the renal system, affects more than 50% of patients in Malaysia [2,5,6], as opposed to 15% in Caucasian populations [7]. In addition, there were substantial complications of SLE, with approximately 40% of Malaysian SLE patients developing organ or system damage [2,8]. However, these findings were derived primarily from single-centre studies with small sample sizes. Therefore, this large, multi-centre study was conducted to validate and consolidate the aforementioned observations.

Remission in SLE with low GC use (≤7.5 mg daily) is associated with a lower probability of damage accrual, as demonstrated by the Systemic Lupus International Collaborating Clinics (SLICC) inception cohort [9] and many other studies [10,11]. Several studies have identified the predictors of treatment response, but the majority of the data were on lupus nephritis [12] involving Caucasian populations [10,13,14]. Given the heterogeneity of SLE and its multi-organ involvement, it was essential to investigate this aspect in a diverse, multi-ethnic SLE population.

## 2. Materials and Methods

### 2.1. Study Design and Population

This was a multicentre retrospective cohort study conducted from September 2023 until October 2024. Systemic Lupus Erythematosus (SLE) patients aged ≥12 years were recruited from the following centres:

(1) Ministry of Higher Education Hospitals: Hospital Canselor Tuanku Muhriz (HCTM) UKM, Universiti Malaya Medical Centre (UMMC), Hospital Universiti Sains Malaysia (HUSM), Universiti Malaysia Sabah Hospital.
(2) Ministry of Health of Malaysia hospitals: Hospital Putrajaya, Kuala Lumpur Hospital, Hospital Tengku Ampuan Rahimah (HTAR Klang), Hospital Selayang, Hospital Sultan Idris Shah Serdang, Hospital Tuanku Ja’afar Seremban, Hospital Melaka, Hospital Raja Permaisuri Bainun (HRPB Ipoh), Hospital Tengku Ampuan Afzan (HTAA, Kuantan), Hospital Sultanah Nur Zahirah (HSNZ, Terengganu), Hospital Raja Perempuan Zainab II (HRPZ II, Kelantan), Hospital Sultanah Bahiyah (Alor Setar), Hospital Pulau Pinang, Sarawak General Hospital, Hospital Sibu, Hospital Queen Elizabeth (Kota Kinabalu), Hospital Tuanku Fauziah (Perlis), Hospital Sultanah Fatimah (Muar).

The centres were divided into 5 regions:

(1) Central of Malaysia: Hospital Canselor Tuanku Muhriz (HCTM) UKM, Universiti Malaya Medical Centre (UMMC), Hospital Putrajaya, Kuala Lumpur Hospital, Hospital Tengku Ampuan Rahimah (HTAR Klang), Hospital Selayang, Hospital Sultan Idris Shah Serdang

(2) West Coast of Malaysia: Hospital Raja Permaisuri Bainun (HRPB Ipoh), Hospital Sultanah Bahiyah (Alor Setar), Hospital Pulau Pinang, Hospital Tuanku Fauziah (Perlis).

(3) East Coast of Malaysia: Hospital Tengku Ampuan Afzan (HTAA, Kuantan), Hospital Sultanah Nur Zahirah (HSNZ, Terengganu), Hospital Raja Perempuan Zainab II (HRPZ II, Kelantan).

(4) Southern of Malaysia: Hospital Tuanku Ja’afar Seremban, Hospital Melaka, Hospital Sultanah Fatimah (Muar).

(5) Borneo: Hospital Queen Elizabeth (Kota Kinabalu), Sarawak General Hospital, Hospital Sibu, Universiti Malaysia Sabah Hospital

A purposive sampling method was used for the subject recruitment.

### 2.2. Inclusion and Exclusion Criteria

**Inclusion criteria:**

- All patients who fulfilled the European League Association of Rheumatology/American College Rheumatology (EULAR/ACR) 2019 classification criteria [15] or Systemic Lupus Erythematosus Collaborating Clinics (SLICC) 2012 classification criteria for SLE [16].
- Had regular follow-up visits (at least 3 visits/year) and completed observation at the same hospital for at least 12 months from diagnosis

**Exclusion criteria:**

- Overlap SLE with other connective tissue diseases, e.g., Rheumatoid Arthritis, Systemic Sclerosis
- Inadequate data or information from the medical records
- Incomplete (<12 months from the time of diagnosis) follow-up at the same hospital.

### 2.3. Consent and Ethics Approval

Research ethics approvals were obtained from the National Medical Research Register (NMRR ID-23-02672-VNL (IIR)) and the UKM Ethics Committee (JEP-2023-590). No consent was obtained from the patients, as this study was limited to the review of medical records.

### 2.4. Sample Size

The sample size is calculated based on the formula for prevalence study (26):

$$n\;=\;{\left(\frac{Z_{1-\alpha /2\;}}{d}\right)}^{2}\left(P\right)\left(1-P\right)$$

n  =  Sample size;

*Z*_1−α/2_  =  Z statistic for a level of confidence (1.96 for 95% confidence level);

P  =  Expected prevalence or proportion;

d  =  Precision.

The prevalence of LLDAS remission is 63.1% [17].

The calculated sample size was 359 from each region, with a total of 1795 subjects.

### 2.5. Data Collection

Section 1: Socio-demographic data and SLE characteristics at diagnosis (baseline)

The information collected included age, gender and ethnicity. SLE characteristics at diagnosis included organ manifestations, immunological parameters (anti-dsDNA and complement [C3 and C4] levels). The age of disease onset was defined as the age at SLE diagnosis. SLE disease duration was defined as the time from SLE diagnosis to the time of data collection. The total SLE disease activity index (total SLEDAI) score was used to categorise the disease severity at diagnosis/baseline [18]:

(1) Mild: SLEDAI < 6

(2) Moderate: SLEDAI 6–12

(3) Severe: SLEDAI > 12

The treatment given at diagnosis was reviewed and the data on the administration of pulse methylprednisolone, maximum dose of oral prednisolone, immunosuppressant(s) and hydroxychloroquine use were recorded.

Section 2. Treatment targets of remission and low disease activity state (LDAS)

Due to the unavailability of Physician Global Assessment (PGA) and serological data (anti-dsDNA, C3 and C4 levels) during follow-up, treatment targets were modified from the validated Definition of Remission in Systemic Lupus Erythematosus (DORIS) [19] and Lupus Low Disease Activity State (LLDAS) [17]. These items were omitted in both remission and low disease activity state (LDAS). Specifically, these parameters were omitted from the criteria for both remission and low disease activity state (LDAS). Consequently, remission was defined according to the 2023 EULAR recommendations as a clinical SLE Disease Activity Index (cSLEDAI) score of 0 with a concomitant oral prednisolone (or equivalent) dose of ≤5 mg/day [18]. LDAS was defined as a cSLEDAI of 0 with an oral prednisolone dose of ≤7.5 mg daily.

In addition to a cSLEDAI score of 0, both conditions required the absence of disease activity in major organ systems (renal, central nervous system, cardiopulmonary, vasculitis, or fever) and no evidence of haemolytic anaemia or gastrointestinal involvement. Combining clinical quiescence (cSLEDAI = 0) with a low-dose steroid limit provided a validated proxy for disease control in retrospective studies lacking comprehensive serological or PGA scoring [20].

A delay in achieving treatment targets was defined as failure to achieve remission or LDAS within 12 months following SLE diagnosis.

### 2.6. Organ or System Damage In SLE

Presence of organ/system damage of SLE (yes/no) that occurred after the SLE diagnosis was recorded based on the Systemic Lupus International Collaborating Clinics/American College of Rheumatology (SLICC/ACR) damage index [21] until the last recorded date of clinic visit.

### 2.7. Statistical Analysis

Continuous variables with normal distribution were reported as mean ± standard deviation (SD) and non-normally distributed continuous variables were reported as median (25–75% interquartile range [IQR]). Categorical variables were reported as frequency and percentage. The normality of the data was assessed using the Kolmogorov–Smirnov test and Q-Q plot. Missing laboratory data, such as antiphospholipid antibodies, anti-dsDNA and complement C3/C4 levels pre-induction treatment, were handled using complete-case analysis. The chi-square test was used for categorical data and Student’s *t*-test for continuous data.

To determine which variables were predictive of delayed remission and damage, variables with *p* < 0.10 in univariable analysis were included in the respective multivariable logistic regression models.

To identify prognostic factors for achieving early SLE treatment targets, the potential prognostic factors were first screened using simple Cox proportional hazards regression, and the variables with *p*-values < 0.25 were selected to develop a multivariable model using multiple Cox proportional hazards regression [22]. Covariate selection for the multiple Cox proportional hazards model was performed manually using a purposeful selection approach, guided by clinical and biological relevance together with *p*-values, with variables added sequentially and removed when not retained in the model [22]. For these procedures, the event was defined as early attainment of SLE treatment targes (remission and LDAS) at 12 months, and the event time was defined as the time from the participant’s entry into the study until the occurrence of early treatment targets of SLE. Participants who did not achieve treatment targets after 12 months or at the time of follow-up at 12 months were considered censored observations, and their times were treated as censoring time. The proportional hazards assumption was assessed objectively using the Grambsch and Therneau test based on scaled Schoenfeld residuals [23] and graphically using plots of Schoenfeld residuals versus time [22].

All data were analysed using SPSS Statistics version 27.0 (IBM Corp., Armonk, NY, USA) and R version 4.52 (R Core Team, Vienna, Austria) via survminer version 0.5.1 and survival version 3.8-3 R packages. A *p*-value of <0.05 was considered statistically significant.

## 3. Results

### 3.1. Baseline Characteristics

At the time of analyses, 1786/1891 SLE patients had at least 1 year of follow-up. A total of 157 patients were excluded due to missing essential data while 30 patients were excluded as they were non-Malaysians. As such, 1599 patients were included in this study. The mean age of this cohort was 37.59 ± 13.32 years with a mean disease duration of 7.55 ± 5.98 years. The mean age of the subjects at the time of diagnosis was 29.85 ± 12.47 years.

The majority of the subjects were of Malay ethnicity (68.7%), followed by Chinese (17.4%), indigenous group (10.8%) and Indians (3%). More than two-thirds of the patients presented initially with mucocutaneous and haematological manifestations. At baseline, anti-dsDNA was positive in 74.8%, while 72.4% and 61.9% had low complement C3 and C4, respectively.

A total of 1567 subjects had total SLEDAI scores recorded at baseline, and the mean SLEDAI score was 10.61 ± 5.80. Approximately half of them (53.5%) presented with moderate disease activity (SLEDAI score 6–12), while 29.6% had high disease activity (SLEDAI score > 12) and 16.8% had low disease activity (SLEDAI score < 6)

The vast majority of the patients (92.3%) were started on hydroxychloroquine at baseline. Approximately 23% of the patients received pulse IV methylprednisolone as the initial treatment and more than one-third received oral prednisolone of 30 mg daily. A total of 50.2% did not receive any immunosuppressant (IS) or steroid-sparing agent as the initial treatment. Azathioprine was the most frequently prescribed initial immunosuppressant (20.9%). The baseline treatment characteristics were summarised in Appendix A.1. No formal statistical analyses were carried out to determine the associations between treatment regimes and outcomes, as the results would be inherently confounded by the indications.

The rates of early remission at 3, 6 and 9 months were 5.9% *(n* = 95), 11.6% (186) and 17.8% (*n* = 285), respectively. Meanwhile, the rates of LDAS at 3, 6 and 9 months were 3.3 (*n* = 53), 5.6% (*n* = 90) and 5.9% (*n* = 94).

### 3.2. Early Remission and the Associated Prognosticators

A total of 730 subjects (45.7%) achieved the treatment targets, of whom 617 (84.5%) achieved early remission. A total of 869 subjects (54.3%) had a delay in achieving the treatment targets of either remission or LDAS. A higher proportion (64.4%) in the indigenous group achieved early remission and LDAS, while only 42.3% of Malay patients achieved the treatment targets (*p* < 0.001). Other factors associated with delayed attainment of remission and LDAS in univariate analyses included longer disease duration and initial manifestations involving haematological, renal and neuropsychiatric lupus. High SLEDAI score, positive anti-dsDNA and low complement(s) were also associated with delayed remission and LDAS. Initiation of hydroxychloroquine at baseline was associated with a lower frequency of delay in achieving remission and LDAS. Delayed attainment of the treatment targets (remission or LDAS) was associated with greater organ damage accrual. Table 1 summarises the univariate analyses of the factors associated with delayed remission in the Malaysian cohort.

To account for the variable time required for each patient to achieve remission, survival analysis was performed using multivariable Cox proportional hazards regression with the time-to-event was censored at 12 months post-diagnosis. In this model, the Malay and Chinese ethnic groups demonstrated a significantly lower likelihood of achieving early remission compared to the Indigenous ethnic group. Furthermore, longer SLE duration, the presence of organ damage, haematological and renal manifestations as the initial presentation and low C4 levels were identified as additional adverse prognosticators associated with lower likelihood of early remission. Table 2 illustrates the simple and multivariable cox regression analysis result.

**Figure A1:**
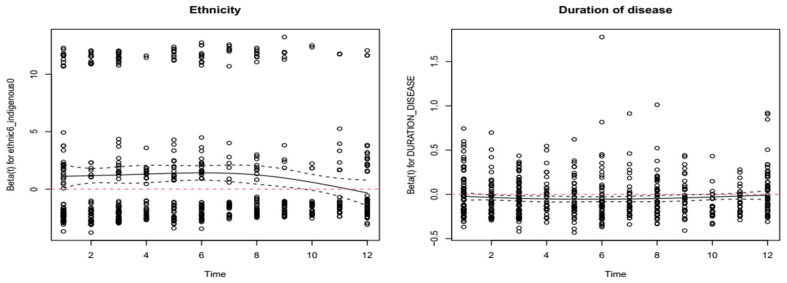

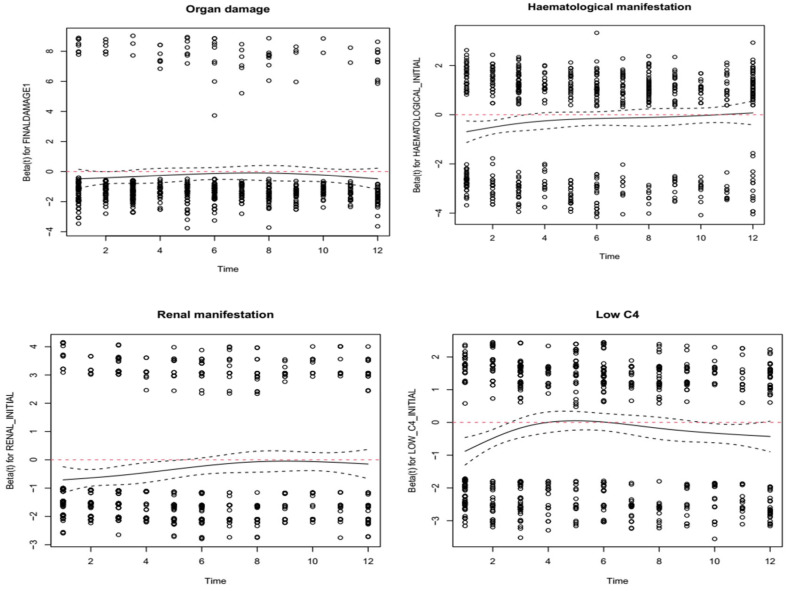
Schoenfeld residual plots versus time for each prognosticator included in the multiple Cox proportional hazards model for early attainment of treatment target in SLE. Open circles denote the scaled Schoenfeld residuals, the solid black curve denotes the smoothed trend, the grey dashed curves denote the approximate 95% confidence bands and the red horizontal dashed line denotes the zero reference.

The proportional hazards assumption was evaluated for the multivariable Cox proportional hazards model for early remission. Based on the Grambsch and Therneau test, haematological manifestations and renal manifestations showed statistically significant results (*p* = 0.003 and *p* = 0.005, respectively), suggesting possible violation of the proportional hazards assumption for these covariates. Nevertheless, inspection of the Schoenfeld residuals versus time plots (Figure A1 in Appendix A.1) demonstrated that the LOWESS curves remained close to zero with no obvious systematic departure over time. Taken together, these findings supported retention of the final Cox model, and no time-varying Cox model was fitted.

### 3.3. Factors Associated with Organ Damage Accrual

Until the last date of follow-up, a total of 270 patients (16.9%) had organ damage. In univariate analysis, factors associated with organ damage were current age, disease duration, positive anti-cardiolipin IgG, renal and NPSLE involvement at baseline. A significantly higher proportion of patients who received intravenous (IV) methylprednisolone and did not receive hydroxychloroquine at baseline accrued organ damage. The use of IS at baseline and oral prednisolone ≥ 7.5 mg at 24 months were also associated with organ damage. Table 3 illustrates the factors associated with organ damage in this SLE cohort.

Further multivariable logistic regression analysis revealed that the independent predictors of damage were not being on HCQ therapy, a delay in achieving the treatment targets (remission and LDAS), disease duration and NPSLE. Indian patients were at risk of organ damage, while Malay ethnicity was protective against damage. Table 4 illustrates the logistic regression analysis of predictors of organ damage in SLE.

## 4. Discussion

Our study found that more than half of the patients failed to achieve treatment targets (remission or LDAS) within the first year of diagnosis. In contrast, early remission rates in predominantly Caucasian cohorts have been reported to range from approximately 50% to 75%. For instance, the Amsterdam SLE cohort reported that 56.2% of patients achieved Lupus Low Disease Activity State (LLDAS) within the first year of follow-up [13]. Similarly, a higher prevalence of LLDAS was observed in an Italian cohort, with rates ranging from 63% to 74.8% at baseline and at each annual evaluation [10]. Findings from a multi-ethnic, multinational Latin American cohort showed that only 20.2% of patients achieved remission and 14.2% achieved LLDAS at least once during a median follow-up period of 2.4 years [24].

There is limited data on early remission from the Asia-Pacific region. A retrospective study of the Iranian SLE cohort demonstrated that more than 50% of active SLE patients achieved remission within 6 months of starting treatment [25]. Meanwhile, a cross-sectional, multinational and multiethnic study involving 1846 patients from the Asia Pacific region, more than 50% of whom were of Chinese ethnicity, reported an LLDAS achievement rate of 44%. Nonetheless, the timing of remission relative to the initial diagnosis was not clearly specified [26]. Malaysia, as a multi-ethnic country with universal healthcare coverage, provides a unique setting to examine these disparities under conditions of equitable healthcare access.

There were significant ethnic disparities in achieving early remission, with the Indigenous group demonstrating a higher likelihood of early remission compared to Malay, Chinese and Indian ethnicities. However, data on remission rates in multi-ethnic cohorts remain scarce, with most published studies focusing on patients with lupus nephritis [12,27,28,29]. The multi-centre, multi-ethnic Aspreva Lupus Management Study (ALMS trial) previously highlighted potential organ-specific differences in treatment responsiveness among patients with lupus nephritis across different racial and ethnic backgrounds. In that study, patients from Hispanic regions were less likely to achieve complete renal response compared to those from Asian regions [12]. The Asia Pacific multi-ethnic SLE cohort did not find any ethnic differences in the attainment of LLDAS [26]. However, it is important to consider that the observed ethnic disparities are likely multifactorial and may reflect differences in the socioeconomic factors and healthcare access, which were not determined in this study.

While ethnic disparities in SLE outcomes are well-documented, existing literature is heavily weighted toward Caucasian and East Asian populations. This study has provided the medical community with critical data on lupus from a highly diverse multi-ethnic Southeast Asian cohort. Furthermore, this research highlighted a potential ‘window of opportunity’ during the first year of diagnosis. Unlike previous studies that assessed disease activity at varying intervals, this study specifically focused on early treatment target attainment, demonstrating that failure to reach these goals within the first 12 months is significantly associated with subsequent organ damage accrual.

In the present study, baseline haematological and renal involvement were significant predictors of delayed achievement of treatment targets. These findings aligned with observations from a multiethnic, multinational Latin American cohort [30]. Meanwhile, a retrospective study of the Italian SLE cohort found that the presence of joint and cutaneous manifestations was associated with a lower likelihood of achieving LLDAS or remission during follow-up. However, the proportions of patients with active lupus nephritis in that cohort were relatively low, at 8% and 0.8%, respectively [10]. The Asia Pacific cohort demonstrated that LLDAS attainment at the time of study were less likely to occur in patients with shorter duration, patients with history of discoid rash, renal disease, elevated anti-dsDNA, hypocomplementaemia and lower national social wealth [26]. It is important to note, however, that both studies assessed disease activity at different time points, whereas our study specifically focused on remission status within the first year following SLE diagnosis.

Most importantly, our study demonstrated that delays in achieving remission and LDAS were significantly associated with the development of organ damage. This finding underscored the importance of a treat-to-target strategy in SLE, particularly within the first year of diagnosis, to prevent subsequent complications. The majority of the studies demonstrated that the duration of sustained remission was associated with a lower risk of organ damage accrual [11,24,31,32,33,34]. Our findings emphasised that timely disease control may offer protective effects against organ damage, as observed in the ALMS trial [12].

Our study also identified ethnic disparities in organ damage accrual. Indian patients were found to have a fivefold increased risk of organ damage compared to other ethnic groups. This finding was parallel to the result from a smaller, single-centre study conducted in Malaysia [2]. Other multi-ethnic studies found that Hispanics and African/Caribbean are at a higher risk of organ damage compared to Caucasian patients [35,36,37]. However, Petri et al. found that the association between Afro-Caribbean ethnicity and damage was no longer significant after multivariable analysis, and this was attributed to other confounding factors such as socio-economic status, hypertension and proteinuria [38], which were not studied in our cohort.

Failure to initiate HCQ therapy at diagnosis was associated with organ damage accrual, highlighting the importance of HCQ therapy [2,30,39,40]. Pulse methylprednisolone, immunosuppressant use at baseline and persistent requirement of oral prednisolone ≥ 7.5 mg daily at 24 months of SLE diagnosis were found to be associated with damage accrual. However, these factors were no longer significant in the multivariable analysis. The association between use of steroids and damage has been widely evaluated in previous reports [2,41,42,43] but the effects of these medications can be confounded by indication bias. More severe disease would require a higher and prolonged steroid therapy. However, few other studies, including those from the Hopkins Lupus, Toronto and Asian cohorts have demonstrated that steroids were still an independent risk factor of damage even after adjustment for disease activity [41,44,45,46].

There were several limitations in this study. As a retrospective cohort study, the analyses were inherently susceptible to selection and information biases, as well as confounding factors that may have influenced the observed ethnic discrepancies, such as education and socioeconomic status, which were not investigated. In addition, the retrospective design did not allow for the temporal sequence between the prognostic factors and the outcomes to be established clearly. Therefore, causality between these factors and the study outcomes, namely damage accrual and early remission, cannot be conclusively inferred. Data on the specific timing of treatment initiation was not collected, precluding the analysis of the association between treatment delay and clinical remission. Furthermore, the lack of Physician Global Assessment (PGA) and complete serological data necessitated the use of modified treatment targets, which may have impacted the precision of our disease state classifications. In addition, the extent of missing data limited the robustness of certain sub-analyses. Hence, these results should be interpreted with caution and should be framed as associations rather than causal explanations.

## 5. Conclusions

In this multi-ethnic Malaysian study, patients presenting with renal involvement, haematological manifestations and low complement C4 were associated with delayed attainment of treatment targets. This study also underlined the importance of a timely treat-to-target approach in preventing or minimising organ damage. Our study revealed the ethnic variations in achieving early remission and organ damage, which warrant further investigation.

## Figures and Tables

**Table 1:** Baseline characteristics and factors associated with delayed remission (*n* = 1599).

Parameters	All (n = 1599)	Early Remission and LDAS, (n = 730)	Delayed Remission and LDAS, (n = 869)	*p*
Current age, years (mean (SD)	37.6 (13.3)	37.9 ± 13.6	37.4 (13.1)	0.50
SLE duration, years (mean (SD)	7.6 (5.9)	6.8 (5.5)	8.4 (6.3)	<0.001
Age SLE diagnosis (baseline age), years (mean (SD))	29.9 (12.5)	30.8 (12.7)	29.0 (12.4)	0.23
Gender, % (*n*)				
Male,	7.5 (119)	7.7 (56)	7.3 (63)	0.78
Female,	92.5 (1474)	92.3 (671)	92.7 (803)	
Ethnic, % (*n*) *				
Malay	68.7 (1096)	63.7 (463)	72.9 (633)	<0.001
Chinese	17.4 (278)	17.6 (128)	17.3 (150)	
Indigenous Sabah	7.3 (117)	10.5 (76)	4.7 (41)	
Indigenous Sarawak	3.3 (53)	4.7 (34)	2.2 (19)	
Indigenous Peninsular	0.2 (3)	0.1 (1)	0.2 (2)	
Indian	3.0 (48)	3.4 (25)	2.6 (23)	
System/organ SLE at baseline, % (*n*)				
Mucocutaneous	73.4 (1173)	73.4 (536)	73.3 (637)	1.00
Haematological	68.0 (1085)	64.1 (467)	71.3 (618)	0.003
Musculoskeletal	64.0 (1022)	64.7 (471)	63.4 (551)	0.40
Constitutional	51.6 (822)	50.1 (365)	52.8 (457)	0.31
Renal	32.8 (524)	26.5 (193)	38.1 (331)	<0.001
Serositis	12.1 (193)	11.9 (87)	12.2 (106)	0.89
Neuropsychiatric	9.1 (146)	7.5 (55)	10.5 (91)	0.04
Baseline SLEDAI score	10.61 ± 5.81	9.9 ± 5.6	11.2 ± 5.9	<0.001
Immunological at baseline				
Anti-dsDNA positive	74.8 (1013)	71.3 (437)	77.6 (576)	0.008
Low C3	72.4 (1032)	66.4 (431)	77.3 (601)	<0.001
Low C4	61.9 (881)	55.6 (359)	67.2 (522)	<0.001
Lupus Anticoagulant positive	31.5 (253)	28.9 (106)	33.8 (147)	0.10
ACL IgG positive	18.5 (162)	18.3 (71)	18.6 (91)	0.93
ACL IgM positive	10.7 (89)	8.1 (29)	12.7 (60)	0.04
Anti B2 GP1 IgG positive	17.5 (115)	15.0 (41)	19.3 (74)	0.18
Anti B2 GP1 IgM positive	10.8 (68)	8.1 (21)	12.6 (47)	0.10
Baseline SLEDAI score	10.61 ± 5.81	9.9 ± 5.6	11.2 ± 5.9	<0.001
Absence of HCQ initial	7.1 (112)	5.5 (40)	8.4 (72)	0.03
Organ damage	16.9 (270)	13.2 (96)	20.0 (174)	<0.001

ACL = anti-cardiolipin, GP1 = glycoprotein-1, LDAS = low disease activity state, HCQ = hydroxychloroquine. * Malay vs. others 72.9 vs. 63.7, *p* < 0.001, All indigenous vs. others 7.2 vs. 15.4, *p* < 0.001.

**Table 2:** Simple and multiple Cox regression analyses of prognostic factors for early remission attainment in SLE. (*n* = 1599).

Parameters	Crude HR^+^ (95% CI)	*p*	Adjusted HR^+^ (95% C.I)	*p*
Ethnicities				
Malay *	0.520 (0.422–0.641)	<0.001	0.576 (0.458–0.724)	<0.001
Chinese *	0.600 (0.465–0.775)	<0.001	0.673 (0.508–0.891)	0.006
Indian *	0.686 (0.444–1.059)	0.089	0.734 (0.456–1.182)	0.204
SLE disease duration	0.965 (0.951–0.979)	<0.001	0.963 (0.947–0.981)	<0.001
Organ damage	0.679 (0.584–0.842)	<0.001	0.755 (0.589–0.968)	0.027
Initial NPSLE	0.741 (0.563–0.976)	0.033	0.812 (0.596–1.105)	0.185 ^a^
Positive Anticardiolipin IgM	0.729 (0.499–1.066)	0.103	0.793 (0.497–1.266)	0.331 ^a^
No HCQ	0.707 (0.514–0.973)	0.033	0.971 (0.676–1.395)	0.875 ^a^
Haematological	0.743 (0.639–0.835)	<0.001	0.790 (0.665–0.938)	0.007
Renal	0.646 (0.548–0.762)	<0.001	0.718 (0.598–0.862)	<0.001
Anti-dsDNA positive	0.780 (0.654–0.929)	0.005	0.902 (0.739–1.102)	0.313 ^a^
Low C3	0.650 (0.552–0.765)	<0.001	0.915 (0.738–1.135)	0.419 ^a^
Low C4	0.683 (0.585–0.798)	<0.001	0.755 (0.640–0.890)	<0.001
SLEDAI score	0.968 (0.955–0.981)	<0.001	0.995 (0.979–1.012)	0.578 ^a^

* compared to the indigenous ethnic group; ^+^ HR: Hazard Ratio; ^a^ *p*-values were obtained based on the manual addition of each variable into the best subset model containing all significant prognosticators.

**Table 3:** Factors associated with organ damage accrual (*n* = 1599).

Parameters	No Damage, (n = 1329, 83.1%)	Organ Damage, (n = 270, 16.9%)	*p*
Current age, years	36.6 ± 12.9	42.4 ± 14.1	<0.001
SLE duration, years	6.9 ± 5.2	10.6 ± 8.2	<0.001
Age SLE diagnosis	29.4 ± 12.2	32.1 ± 13.6	0.001
Gender, *n* = 1593			
Male, % (*n*)	6.9 (92)	10.1 (27)	0.09
Female, % (*n*)	93.1 (1233)	89.9 (241)	
Ethnic, % (*n* = 1595)			
Malay	70.7 (937)	58.9 (159)	<0.001
Chinese	15.4 (204)	27.4 (74)	
Indigenous Sabah	8.0 (106)	4.1 (11)	
Indigenous Sarawak	3.0 (40)	4.8 (13)	
Indigenous Peninsular	0.2 (2)	0.4 (1)	
Indian	2.7 (36)	4.4 (12)	
System/organ SLE, % (*n*)			
Mucocutaneous	74.0 (984)	70.0 (189)	0.18
Haematological	73.8 (899)	69.1 (186)	0.72
Musculoskeletal	64.6 (857)	61.1 (165)	0.29
Constitutional	49.4 (350)	53.3 (472)	0.12
Renal	30.6 (406)	43.7 (118)	<0.001
Serositis	12.4 (165)	10.4 (28)	0.41
Neuropsychiatric	7.8 (103)	16.0 (43)	<0.001
Baseline SLEDAI score	10.5 ± 5.8	11.2 ± 6.1	0.07
Immunological at onset (baseline)			
Anti-dsDNA positive	75.3 (850)	72.1 (163)	0.32
Low C3	71.9 (859)	74.9 (173)	0.38
Low C4	61.8 (739)	62.3 (142)	0.94
Lupus Anticoagulant positive	30.6 (208)	36.6 (45)	0.21
ACL IgG positive	16.9 (124)	27.0 (38)	0.006
ACL IgM positive	10.0 (70)	14.6 (19)	0.12
Anti B2 GP1 IgG positive	16.9 (96)	21.1 (19)	0.37
Anti B2 GP1 IgM positive	10.2 (56)	14.1 (12)	0.26
Treatment at baseline			
Absence of HCQ	5.6 (74)	14.3 (38)	<0.001
Pulse methylprednisolone	23.5 (216)	36.4 (60)	<0.001
Prednisolone > 30 mg/day	39.8 (521)	38.3 (101)	0.79
Prednisolone > 7.5 mg baseline	87.3 (1142)	88.6 (234)	0.61
Prednisolone > 7.5 mg at 24 months	26.1 (251)	34.1 (70)	0.03
Any immunosuppressant	48.4 (641)	59.5 (157)	<0.001

ACL = anti-cardiolipin, HCQ = hydroxychloroquine.

**Table 4:** Multivariable logistic regression of the predictor(s) of damage accrual (*n* = 1599).

Parameters	B Coefficient	Adjusted OR (95% C.I)	*p*
Disease duration	0.10	1.11 (0.99–1.23)	0.05
Delay remission or LDAS	1.09	2.97 (1.51–5.83)	0.002
Not on hydroxychloroquine ^£^	1.42	4.13 (1.21–14.07)	0.02
Malay ^#^	−0.72	0.48 (0.25–0.94)	0.03
Indian *^&^	1.75	5.75 (1.39–23.81)	0.02
Chinese *	0.44	1.55 (0.71–3.39)	0.27
Indigenous *	0.97	2.64 (0.78–9.02)	0.12
NPSLE	0.72	2.06 (0.81–5.26)	0.13
Current age	−0.01	0.99 (0.91–1.10)	1.08
Age of SLE diagnosis	0.07	1.07 (0.98–1.16)	0.13
Male	−0.09	0.92 (0.23–3.66)	0.90
Initial aCL IgG positive	0.47	1.60 (0.44–3.45)	0.23
Renal	−0.03	0.98 (0.46–2.09)	0.95
Baseline SLEDAI score	0.02	1.02 (0.96–1.08)	0.58
Pulse methylprednisolone at baseline	0.25	1.29 (0.62–2.68)	0.50
Prednisolone ≥ 7.5 mg at 24 months	0.29	1.34 (0.47–3.78)	0.90
Any immunosuppressant (IS) ^£^	−0.08	0.93 (0.46–1.86)	0.84

* vs. Malay. ^#^ Malay vs. other ethnics. ^&^ Indian vs. other ethnics: 5.73 (1.27–25.96), *p* = 0.02. ^£^ At baseline or initial.

## Data Availability

Data is available in Supplementary File S1.

## Supplementary Materials

The following supporting information can be downloaded at: https://www.mdpi.com/article/10.3390/jcm15093387/s1, Supplementary File S1.

## Abbreviations

The following abbreviations are used in this manuscript:

ACL	Anti-cardiolipin
CI	Confidence Interval
cSLEDAI	clinical SLE Disease Activity Index
DORIS	Definition of Remission in SLE
GC	glucocorticoid
GP1	glycoprotein-1
EULAR/ACR	European League Association of Rheumatology/American College Rheumatology
LLDAS	low lupus disease activity state
HCQ	Hydroxychloroquine
IS	Immunosuppressant
NPSLE	Neuropsychiatric lupus
OR	Odds ratio
SLE	Systemic lupus erythematosus
SLICC	Systemic Lupus International Collaborating Clinics

## Appendix A

### Appendix A.1

**Table A1:** Baseline treatments received by the SLE cohort (*n* = 1599).

**Type of Treatment (n)**	**N (%)**
Pulse methylprednisolone ≥ 250 mg	365 (22.8)
Prednisolone dose (mg/day)	
0	120 (7.5)
≤5 mg	63 (3.9)
6–10	145 (9.1)
11–20	257 (16.1)
21–30	365 (22.8)
31–60	604 (37.8)
>60	18 (1.1)
Unknown	27 (1.7)
Hydroxychloroquine	1476 (92.3)
Immunosuppressant (IS) at baseline	
None	809 (50.6)
Azathioprine	336 (21.0)
Cyclophosphamide	242 (15.1)
Mycophenolate	101 (6.3)
Methotrexate	77 (4.8)
Calcineurin inhibitors	11 (0.7)
Other IS	13 (0.8)
unknown	11 (0.7)
Other treatment:	
Rituximab	7 (0.4)
Plasmapharesis/exchange	47 (2.9)
Intravenous immunoglobulin	88 (5.5)
